# Evolving in islands of mud: old and structured hidden diversity in an endemic freshwater crayfish from the Chilean hotspot

**DOI:** 10.1038/s41598-021-88019-8

**Published:** 2021-04-21

**Authors:** Pedro F. Victoriano, Guillermo D’Elía

**Affiliations:** 1 Departamento de Zoología. Facultad de Ciencias Naturales y Oceanográficas, Universidad de Concepción, Casilla 160-C, Concepción, Chile; 2 Instituto de Ciencias Ambientales y Evolutivas, Facultad de Ciencias, Universidad Austral de Chile, Campus Isla Teja S/N, Valdivia, Chile

**Keywords:** Evolution, Population genetics

## Abstract

*Parastacus* is a genus of South American freshwater crayfishes disjunctively distributed in southern Chile, Northern Argentina, Uruguay and Southeastern Brazil. *Parastacus pugnax* is a Chilean endemic distributed along 700 km of latitude in central-southern Chile from the Pacific coast to the Andean piedmont, which is intensively captured for consumption for local communities. Considering the habitat (wet meadows) and natural history (primary burrower, non-migrant) of the species, we tested a hypothesis of highly structured genetic diversity using mtDNA of 465 specimens gathered at 56 localities across the species range. The crown age of *P. pugnax* was estimated at 38 Ma, predating the main Andean uplift. The genetic variation of *P. pugnax* is large and geographically structured. In some cases, genetic groups do not match basin limits, suggesting a previous to current dynamic of basin evolution. The uncovered intraspecific main lineages have different demographic histories. A latitudinal cline in past effective population size reduction suggests environmental singularities with a glacial effect in the southern populations. We suggest adding morphologic and more genetic data in order to assess species limits. Our results contribute to improve future conservation actions for this taxon, providing basic information to delimit conservation units.

## Introduction

Inquiring into the historical processes that have affected genetic diversification is essential to build solid approaches to explain current geographic patterns of intraspecific biodiversity^1,2^. Population differentiation and geographic arrangements of genetic diversity are influenced by many factors, including population age, historical population sizes, level of gene flow, selection regimes, and the history of landscape^3^. Current spatial patterns of genetic variation have largely resulted from the role played by habitat discontinuities e.g.^4^ and the historical stability of habitats, which in turn is influenced by episodes of past climate change^5,6^.

Western temperate South America is characterized by a complex geological history, with accentuated mountain uplifts, volcanism, marine transgressions, and recurrent climatic changes. The beginning of the orogenic processes in the Pacific coasts of temperate South America date back to the Paleozoic, when the Coastal range started its uplift^7^. Meanwhile, the Andean uplift was accentuated about 23 million years ago; the relief with heights similar to present ones was reached about 5 million years ago (see physiographic units in Fig. S1). Although the main barrier effect of the austral Andes was the biogeographic and climatic divorce between the eastern and the Chilean slopes, in the Chilean side it also added a high roughness, conforming many river basins and transverse ranges, which for many species represent numerous interrupted habitats, distributed along a steep climatic gradient. Over these complex landscapes, several glacial events took place from the latest Miocene (c. 6 Ma)^8–10^. There is phylogeographical evidence of the high impact of these events, including population fragmentation, local extinction and population expansion e.g.,^11–20^, in the intraspecific pattern of variation for several taxa, both aquatic and terrestrial.

Species inhabiting superficial freshwater show distributions restricted by the limits of aquatic drainages and usually show patterns of genetic structure consistent with watershed boundaries e.g.,^21,22^. However, species inhabiting non-running subterranean waters have been much less studied. This is the case of burrowing freshwater crayfishes, which inhabit specific kinds of soils, generally associated to wet meadows (clayey sedimentary areas), often not connected to main watercourses, and which are generally highly fragmented and dependent on underground aquifers^23^.

The family Parastacidae (ca. 200 species) is a group present in South America, Madagascar, Australia, Tasmania, New Guinea, and New Zealand^24^, which diverged from its Laurasian counterpart in the Triassic, after the breakup of Pangea^25^. South American parastacids form a monophyletic group^26,27^ with a disjunct distribution. The largest phylogenetic diversity is concentrated in Chile where the three South American genera (*Parastacus, Virilastacus*, and *Samastacus*) are present, summing seven species. Then, 13 species of *Parastacus* are distributed within eastern South America in southeastern Brazil, northeastern Argentina, and Uruguay^28^. This distributional pattern suggests that the basal radiation of *Parastacus* predates the Andean uplift.

**Figure 1:**
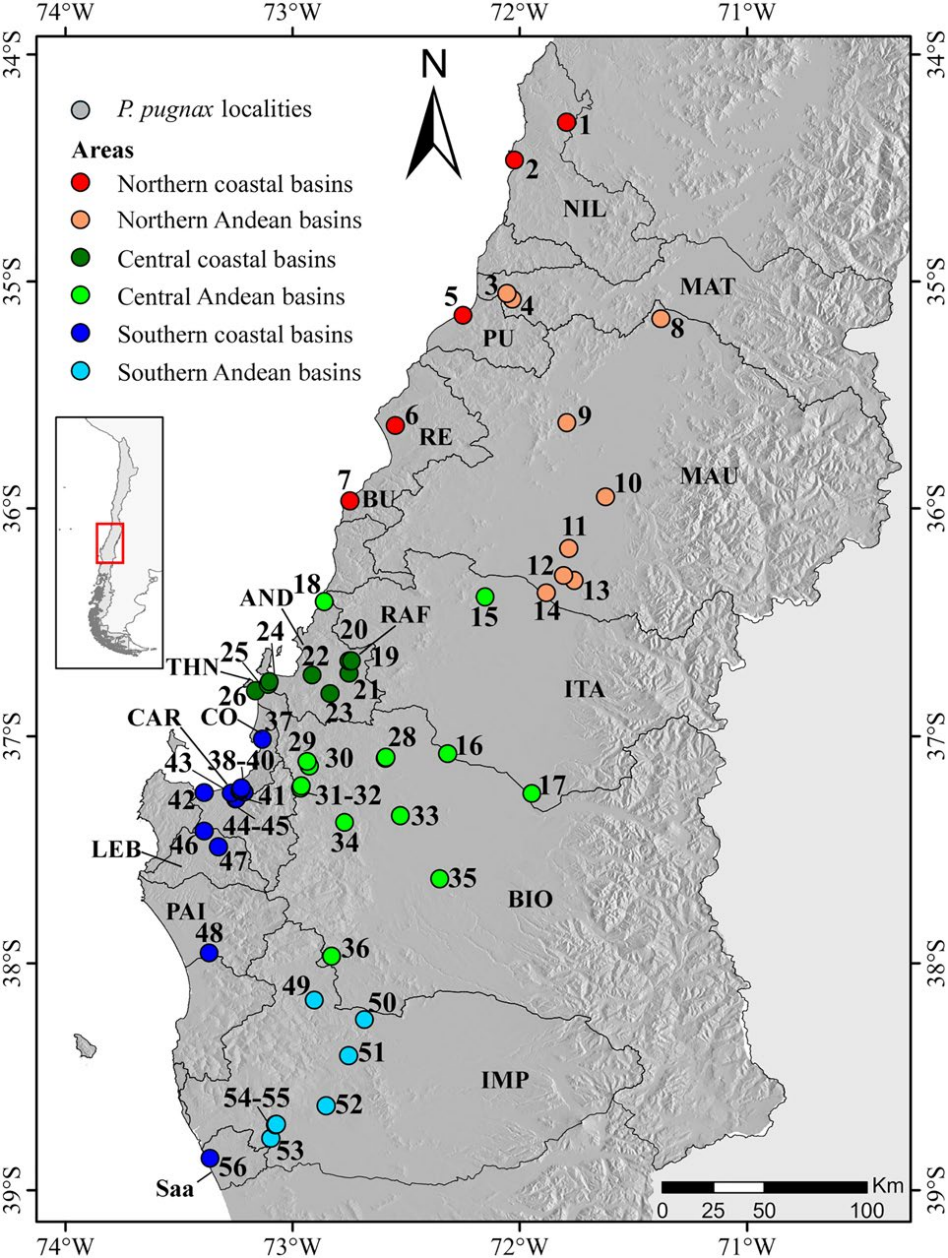
Sampling sites of *Parastacus pugnax*. Localities numbers correspond to those detailed in Table 1. Colors indicate the bioclimatic− hydrographic zones: red + orange correspond to Sub-humid Mediterranean; green + light green the Wet Mediterranean area; and blue + light blue the Per-humid Mediterranean area. Acronyms correspond to basins as described in Table 1. Physiographic units are described in Fig. S1. The map was generated with QGIS v.3.16.2 (https://qgis.org/es/site/) and modified using Inkscape v.0.91 (http://inkscape.org/).

In Chile *Parastacus* is represented by two endemic species^29^; see also^30^. *Parastacus nicoleti* (Philippi 1882) is distributed in the Coastal range of the Valdivian forest from the Tolten basin (ca. 39° S) to Hueyusca, Los Lagos, in the Río Bueno Basin (ca. 40° 55′ S). The second species is *P. pugnax* (Poeppig, 1835), the focal species of this study, which is parapatrically distributed to the north of *P. nicoleti*; it ranges from Nehuentue in La Araucania (ca. 39° S) to the Aconcagua river in Valparaiso (ca. 32° S), throughout a varied range of climates and a heterogeneous physiography (Fig. 1). Latitudinally (ca. 700 km), the range of *P. pugnax* shows an increase in rainfall and a decrease in average temperatures from north to south. *Parastacus pugnax* is a primary burrower that permanently inhabits subterranean waters. It lives mainly in wet meadows, locally known as ¨vegas¨, from which its vernacular name, ¨camarón de vega¨, derives. It is also a species of great cultural value; it is used as a source of food and is massively harvested for local communities every winter^31^. No study has assessed the level of morphologic or genetic variation of *P. pugnax*. Here we start filling this gap by presenting the first assessment of the genetic variation of this species.

Given this information, we expect that *P. pugnax* shows highly divergent intraspecific lineages, likely structured according to hydrographic limits. Additionally, as glaciations have occurred repeatedly since the late Miocene, generating large demographic and distributional changes on the biota of southern South America^32^, events of population expansion are also expected for *P. pugnax*. Accordingly, the objective of this study is to assess the genetic variation in *P. pugnax* throughout its distributional range and to assess if historical climate and geographic factors explain the detected pattern.

## Results

### Sequence data and genealogies

We obtained 465 sequences of the mitochondrial COI gene of 629 base pairs (bp), which resulted in 168 different haplotypes, in which 186 positions (29.57%) were variable. The result of the Xia test indicated low saturation levels in the set of COI sequences (Iss < Iss.c). The average haplotypic diversity (Hd) and the average nucleotide diversity (pi) were 0.98 and 0.061 respectively. Detailed values for genetic variation per locality are shown in Table 1. Although Hd values did not show a clear geographical trend, pi values tended to decrease toward the southern basins. Most of the localities showed private haplotypes; the few haplotypes shared among localities occurred in south central areas (e.g., among Biobío basin localities HU, CHA, YU and Yum; in the southern coastal area of the Carampangue basin among localities LM, CR, VC, PC, P1, HO; see locality acronyms in Table 1). The p-distance values between locality pairs were positively and significantly correlated with geographical distances (Fig. S2); however, although the correlation was significant, in many comparisons between closely locations, both high and low values of genetic distances were found. The highest values of genetic distance were for comparisons between southern basins (Saavedra (Saa) and Imperial (IMP) basins) and localities of center and southern areas (e.g., Talcahuano (THN) v/s Imperial (IMP), 0.091; Talcahuano (THN) v/s Saavedra (Saa), 0.095; Nilahue (NIL) v/s Saavedra (Saa), 0.089, Table S2). Similarly, relatively low values ​​of genetic distance were estimated for some comparisons between Andean v/s central coastal basins (e.g., Maule-Rafael, 0.011). Basin size shows a positive and significant association with pi (Rho = 0.621; p < 0.001; Fig. S3), while the association with Hd was not significant.

**Table 1:** Sampling localities of specimens of *Parastacus pugnax*.

	Locality	Basin	Code	Latitude	Longitude	N	H	Hd	Pi	Fu's F
1	Pailimo		PA	− 34.300	− 71.792	3	3	1.000	0.0088	− 0.076
2	Cahuil		CA	− 34.466	− 72.021	2	2	1.000	0.0352	2.639
		Nilahue	NIL			5	5	1.000	0.0200	− 0.714
3	Curepto 1		CU1	− 35.054	− 72.057	9	5	0.933	0.0128	− 1.727
4	Curepto 2		CU2	− 35.080	− 72.031	9	7	0.833	0.0049	0.126
		**Mataquito**	MAT			18	9	0.947	0.0086	− 5.847 **
5	Putu	Putu	PU	− 35,151	− 72.248	10	3	0.600	0.0012	− 0.101
6	Reloca	Reloca	RE	− 35,637	− 72.547	5	3	0.700	0.0026	0.276
7	Buchupureo	Buchupureo	BU	− 35,968	− 72.747	10	6	0.778	0.0019	− 3.293 **
8	Molina		MO	− 35.166	− 71.377	10	1	–	–	–
9	Alquihue		AL	− 35.623	− 71.791	9	2	0.389	0.0005	0.477
10	Longavi		Long	− 35.950	− 71.621	10	6	0.844	0.0062	− 0.628
11	El Manzanal		PAR	− 36.177	− 71.783	5	3	0.800	0.0022	0.061
12	San Gregorio1		SG1	− 36.296	− 71.805	5	5	1.000	0.0187	− 0.122
13	San Gregorio2		SG2	− 36.318	− 71.758	8	6	0.929	0.0055	− 1.500
14	Buli Caserio		BC	− 36.373	− 71.883	8	4	0.750	0.0022	− 0.518
		**Maule**	MAU			55	24	0.941	0.0277	− 1.174
15	Verquico		VE	− 36.389	− 72.150	3	3	1.000	0.0095	0.587
16	Charrua		CHA	− 37.079	− 72.317	10	2	0.200	0.0041	4.860
17	Tucapel		TU	− 37.254	− 71.946	8	5	0.786	0.0026	− 1.448
18	Vega Itata		VI	− 36.410	− 72.860	8	6	0.929	0.0034	− 2.175 *
		**Itata**	ITA			34	17	0.894	0.0389	3.575
19	Rafael 1		RA1	− 36.669	− 72.739	10	1	–	–	–
20	Rafael 2		RA2	− 36.671	− 72,751	10	1	–	–	–
21	El Carmen		EC	− 36.725	− 72.751	5	4	0.900	0.0046	− 0.278
		Rafael	RAF			25	5	0.363	0.0017	− 0.444
22	Penco		PE	− 36.732	− 72,912	10	4	0.644	0.0032	0.783
23	Florida 1		FL	− 36.815	− 72.834	10	6	0.867	0.0160	2.326
		Andalien	AND			47	10	0.884	0.0206	1.972
24	Denavi Sur		DS	− 36.760	− 73.101	5	3	0.700	0.0017	− 0.185
25	Talcahuano		TH	− 36.774	− 73.107	10	3	0.600	0.0017	0.837
26	Hualpen		HU	− 36.801	− 73.162	10	2	0.200	0.0101	9.186
		Talcahuano	THN			25	3	0.470	0.0054	2.984
27	Yumbel1		YU	− 37.097	− 72.591	10	1	–	–	–
28	Yumbel 2		Yum	− 37.094	− 72.589	10	4	0.533	0.0240	7.436
29	Unihue 1		UN1	− 37.114	− 72.935	10	3	0.644	0.0017	0.390
30	Unihue 2		UN2	− 37.133	− 72.925	5	1	–	–	–
31	Curali Bajo		CB	− 37.220	− 72.959	10	4	0.644	0.0011	− 3.158 **
32	Curali		Sta	− 37.230	− 72.963	10	5	0.667	0.0014	− 2.259 **
33	Santa Elena		Laj	− 37.351	− 72.524	10	3	0.378	0.0152	7.844
34	Nacimiento		NC	− 37.382	− 72.771	2	2	1.000	0.0061	1.386
35	Mulchen		MU	− 37.630	− 72,352	10	3	0.378	0.0002	− 0.339
36	Los Sauces		LS	− 37.970	− 72.827	10	7	0.911	0.0036	− 2.814 *
		**Biobío**	BIO			91	34	0.949	0.0625	8.144
37	Coronel	Coronel	CO	− 37.015	− 73.131	10	1	–	–	–
38	Vega Chillancito		VC	− 37.229	− 73.224	10	1	–	–	–
39	Horcones		HO	− 37.237	− 73.228	9	5	0.806	0.0009	− 0.532
40	Los Maitenes		LM	− 37.241	− 73.231	10	4	0.533	0.0005	− 1.163 *
41	Pichilo		PI	− 37.249	− 73.214	7	3	0.524	0.0008	− 0.922
42	Camino a Llico		LL	− 37.250	− 73.389	9	2	0.222	0.0006	− 0.532
43	Punta Carampangue		PC	− 37.252	− 73.269	8	2	0.571	0.0008	0.866
44	Arauco (Vega Weisser)		Ara	− 37.278	− 73.250	10	2	0.200	0.0009	− 1.163 *
45	Carampangue		CR2	− 37.276	− 73.246	9	2	0.500	0.0007	0.849
		Carampangue	CAR			72	8	0.633	0.0012	− 3.365
46	Curanilahue 1		CUR1	− 37.420	− 73.388	10	2	0.222	0.0003	− 2.369**
47	Curanilahue 2		CUR2	− 37.489	− 73.326	5	3	0.800	0.0015	− 0.475
		Lebu	LEB			15	5	0.714	0.0004	− 7.787**
48	Huentelolen	Paicavi	PAI	− 37.957	− 73.365	7	1	–	–	–
49	Lumaco		Luma	− 38.164	− 72.903	10	7	0.944	0.0036	− 6.867 **
50	Traiguen		Trai	− 38.250	− 72.682	10	5	0.756	0.0022	− 3.003 **
51	Galvarino		GA	− 38.408	− 72.753	9	2	0.389	0.0005	− 0.107
52	CholChol		CHO	− 38.629	− 72.851	4	3	0.833	0.0077	1.098
53	Rulo		RU	− 38.773	− 73.095	8	3	0.607	0.0010	− 0.477
54	Carahue		Car	− 38.710	− 73,070	10	4	0.711	0.0013	− 1.020
55	Imperialito		IM	− 38.713	− 73.075	10	3	0.600	0.0007	1.029
		**Imperial**	IMP			61	25	0.961	0.0227	− 1.667
56	Puerto Saavedra	Saavedra	Saa	− 38.861	− 73.361	10	5	0.667	0.0016	− 2.847 *

Localities are presented from north to south and are grouped by river basin. Andean basins are indicated in bold. N, sample sizes; H, number of haplotypes; Hd, haplotype diversity; Pi, nucleotide diversity; Fs, Fu´s demographic statistics. Statistically significant values (* p < 0.05; ** p < 0.001) are shown in bold.

**Figure 2:**
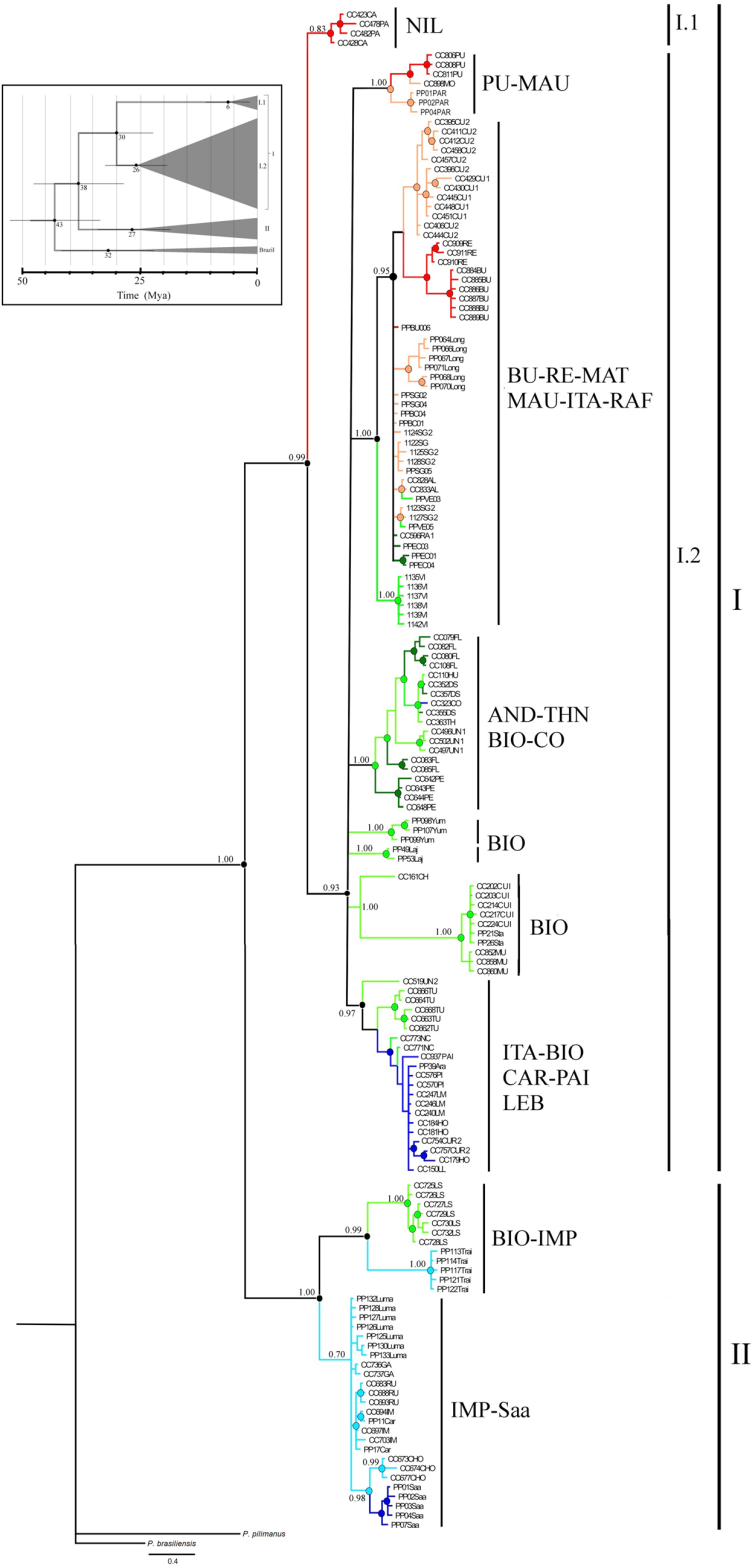
Genealogical relationships of COI *haplotype*s of *Parastacus pugnax* as inferred by Bayesian analysis. Colors correspond to those depicted in Fig. 1. Bayesian a posteriori probabilities (PP) values are given only for the main clades; circles on nodes indicate PP values equal or higher than 0.8. Acronyms correspond to basins as described in Table 1. The box at the top indicates the divergence times (in million years) for the main clades.

Haplotypes of *P. pugnax* form a highly supported clade (PP = 1.0). The Bayesian tree showed a latitudinally structured topology, with three main genealogical groups. The basal dichotomy of the gene tree leads to two main clades, which are allopatric (Fig. 2). The most widely distributed clade (Clade I; PP = 0.99; Fig. 2) includes haplotypes recovered from the northern and central distribution range of the species, from the Nilahue basin in the north, to the Biobío and Paicaví basins in the south. The second clade (Clade II; PP = 1.0) includes haplotypes from the southern fraction of the species range, including basins (IMP and Saa) laying south of the BioBío basin and variants from one locality (Los Sauces) from the Biobío basin, which lays at the limit of the Biobío and Imperial basins. Within the large northern-central clade I, two main subclades were recovered. Clade I.1 (PP = 0.83) exclusively includes haplotypes of the coastal Nilahue basin (NIL) in the northernmost distribution of *P. pugnax*, while clade I.2 (PP = 0.93) is widely distributed and includes haplotypes distributed from the northern Andean Mataquito basin to the coastal Paicaví basin in the south. Within clade I.2 Andean basins showed relationships with several coastal basins, and no reciprocal monophyly was detected at the level of such basins. For example, a southern clade within clade I.2 included some Andean haplotypes both from the Itata and the Biobío rivers, and coastal haplotypes from the coastal Carampangue basin. Within the southern clade II there are three highly divergent subclades; one corresponding to variants from locality 36 of Los Sauces (at the southern edge of the Biobío basin), a second including haplotypes from specimens collected at Traiguén (locality 50), and the third one includes sequences from the rest of the Imperial basin and the coastal site of Puerto Saavedra (Fig. 2). The average values of genetic distance among the three main clades (I.1, I.2, and II) were relatively high (p-distances I.1 v/s I.2 = 0.070; I.1 v/s II = 0.086; I.2 v/s II = 0.102). The crown age of *P. pugnax* was estimated at 38 Mya, during the late Eocene. Crown age of clade I was estimated at 30 Mya ago; while within this, crown age of the northern clade I.1 was estimated at ca. 6 Mya and that of the central clade I.2 was estimated at ca. 26 Mya. Finally, crown age of southern clade II was estimated at 27 Mya (Fig. 2).

**Figure 3:**
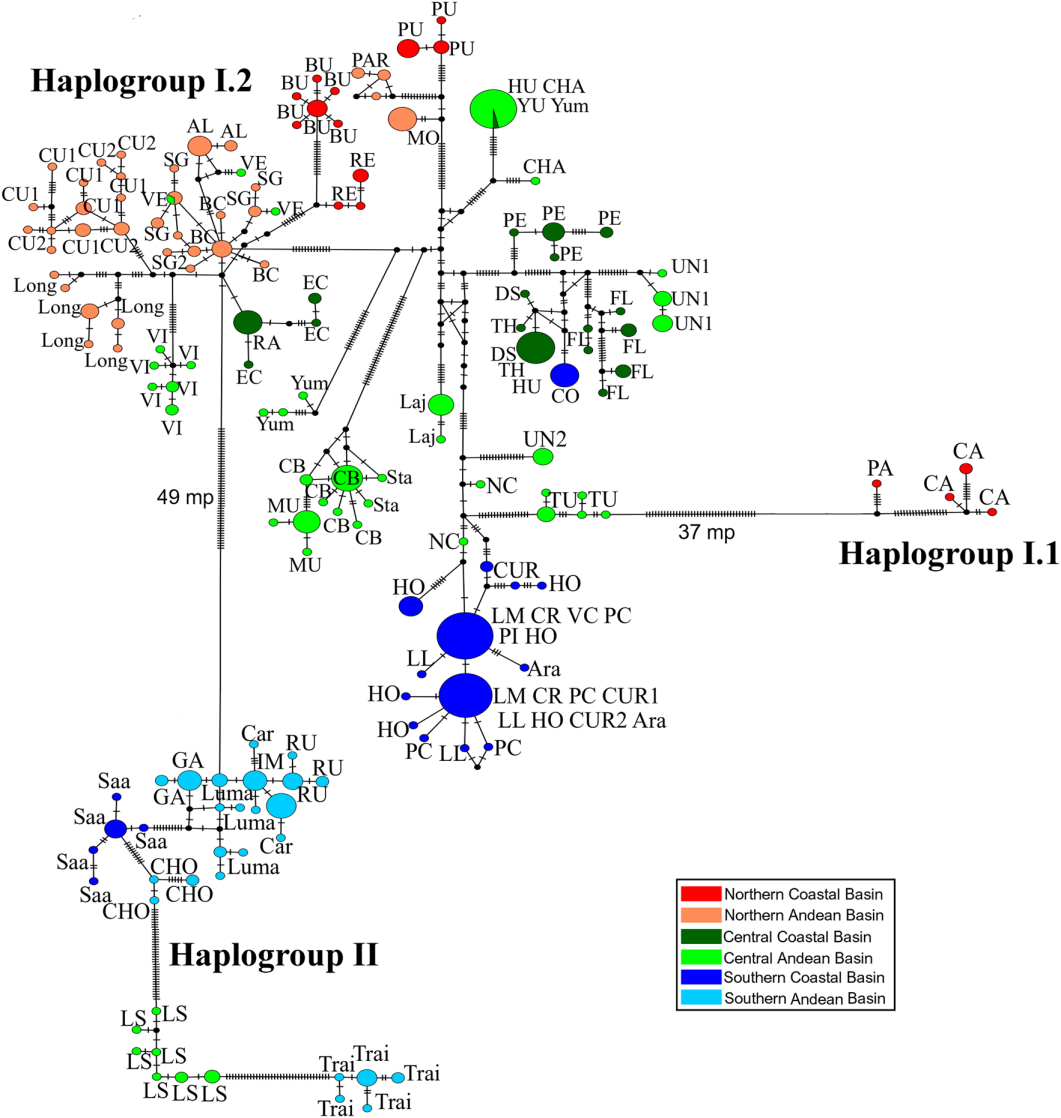
Haplotype network of mitochondrial sequences (COI) of *Parastacus pugnax*. Colors correspond to bioclimatic-hydrographic zones as depicted in Fig. 1. Acronyms correspond to localities as described in Table 1.

The haplotype network showed three highly divergent main haplogroups (haplogroups I.1, I.2 and II; Fig. 3) that correspond to the main clades (I.1, I.2, and II) found in the gene tree. The northern haplogroup I.1 is separated from the large central haplogroup I.2 by 37 mutational steps, while the southern haplogroup II connects to the central I.2 with a branch of 49 mutational steps. Although the three main haplogroups are well defined by their large number of mutational differences, variants of some basins do not form exclusive haplogroups, which was consistent with the existence of polytomies detected in the gene tree.

### Genetic structure

**Figure 4:**
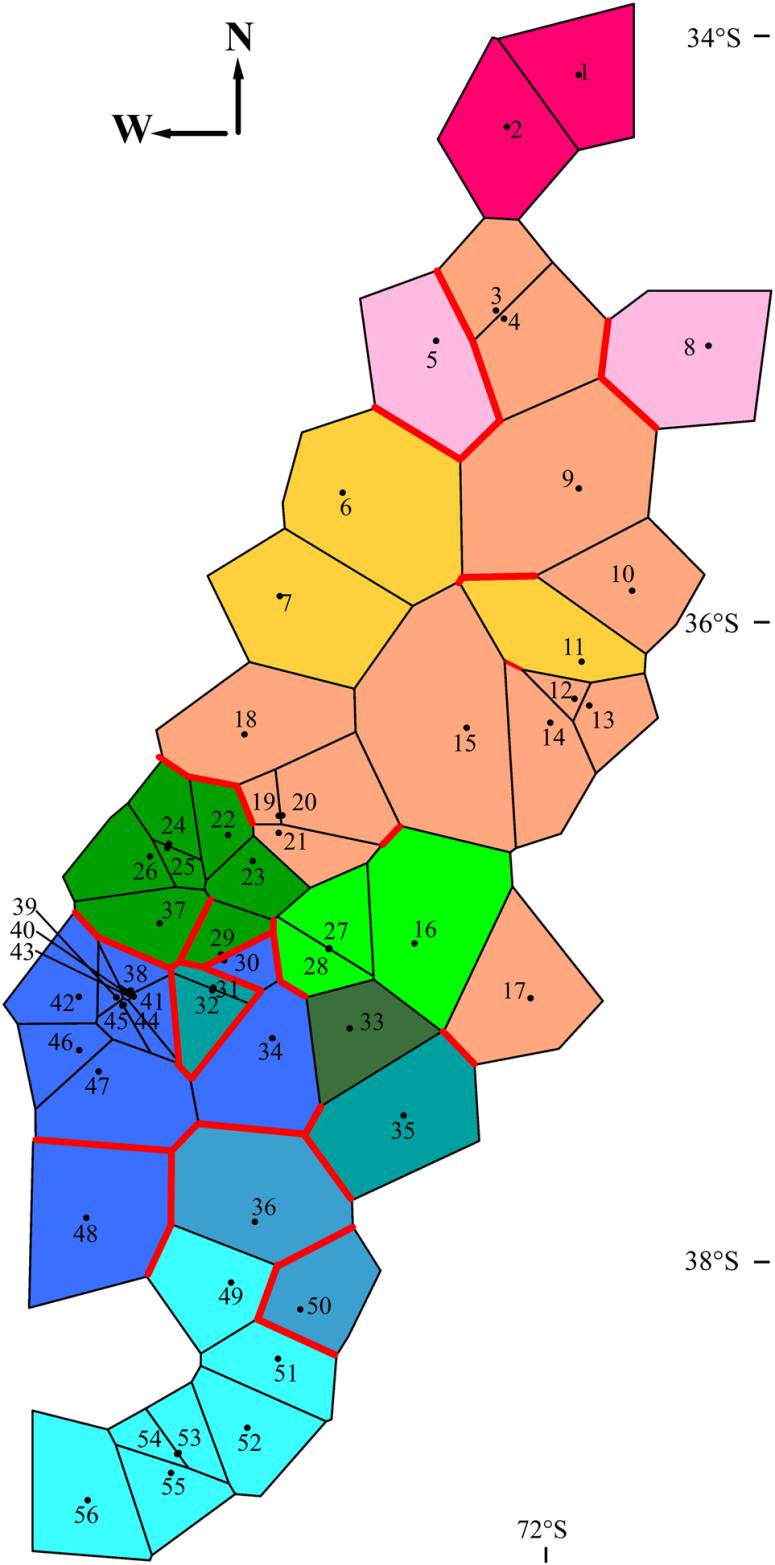
Genetic clusters (obtained from SAMOVA) and barriers based on the distribution of the genetic variation of the COI gene of *Parastacus pugnax*. Colors distinguish each cluster. Numbers correspond to each locality as indicated in Table 1. The red lines indicate the barriers determined by BARRIER 2.2.

All Fst values for comparisons between basin pairs were significant and a large proportion of the comparisons showed a high level of structuring (ca. 40% of inter-basins comparisons with values greater than 0.9; Table 2). This trend was clearly more frequent in comparisons involving coastal basins, while some comparisons among Andean and non-Andean basins tended to show lower levels of differentiation. For example, three of the large Andean basins (Maule, Itata, and Biobío) generally showed less genetic differentiation, both among them and with respect to the coastal basins. In fact, the lowest values were for the Maule-Rafael (Fst = 0.155), Itata-Biobío (Fst = 0.182) and Maule-Itata (Fst = 0.221) comparisons. The spatial analysis of molecular variance (SAMOVA) delimited 11 distinct genetic clusters (FCT = 0.764; p < 0.001; Fig. 4). In general, limits of clusters were not according to basins. In addition, groups tend to present smaller distributions toward the south and north of the species range, while in the central part of the distribution of *Parastacus pugnax* there was one largely distributed cluster (formed by localities 3, 4, 9, 10, 12–15, 17–21; Fig. 4). Many of the localities that form this large central cluster are from the Andean basins of Mataquito, Maule, and Itata. Meanwhile, BARRIER 2.2 identified several putative gene flow barriers that are partly consistent with limits among the clusters delimited with SAMOVA (Fig. 4) throughout the distribution of *P. pugnax*. Of all tessellation boundaries, 35 were suggested as important barriers, which tend to be distributed in the northern and southern ranges of the range of *P. pugnax*. A series of 6 barriers separate localities distributed approximately from 35° S to 36° S. On the other hand, in the south, a high density of barriers is found. One set of barriers separate part of Biobío from some coastal basins (ca. 37° S). Another set of barriers suggests low historical gene flow between Biobío, Imperial and southern coastal basins (ca. 38° S). From the AMOVA, the values of genetic differentiation were significant for the three partitions (p < 0.001), suggesting high structuring at geographic scales, but the largest fraction of the genetic variation corresponds to comparisons among groups (basins, 53.27%). The lowest genetic variation was for the within localities hierarchy (5.26%; Table 3).

**Table 2:**
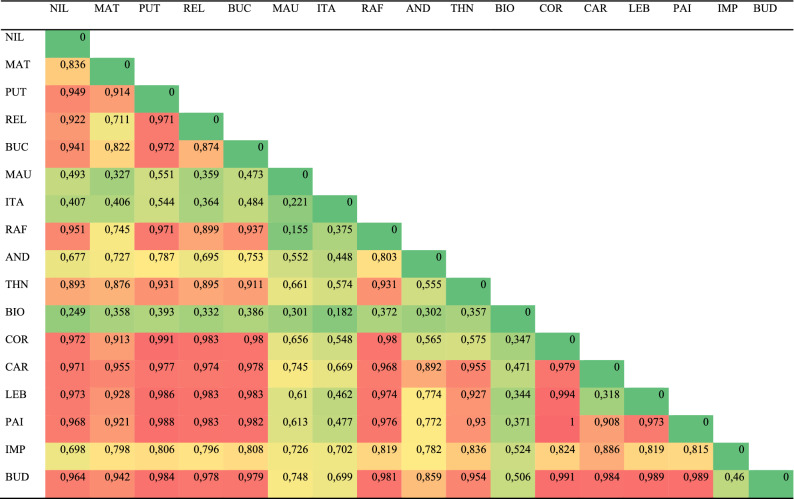
Estimated Fst values for pairwise comparison between basins, throughout the distribution of *P. pugnax*. Basin codes as in Fig. 1 and Table 1. Basins are ordered latitudinally (North to the left). Green: lowest values; red: highest values. All Fst values are significant (p &; 0.001).

**Table 3:** Analysis of molecular variance (AMOVA) of *Parastacus pugnax* populations, considering the basins as groups and localities as populations.

Source of variation	d.f	Sum of squares	Variance components	Percentage of variation
Among groups (basins)	14	5074.667	9.78334 Va	50.66
Among populations within groups	41	2906.002	8.51379 Vb	44.08
Within populations	409	415.420	1.01570 Vc	5.26

The three hierarchical level of variation showed significant structuring (p < 0.001).

### Historical demographic changes

**Figure 5:**
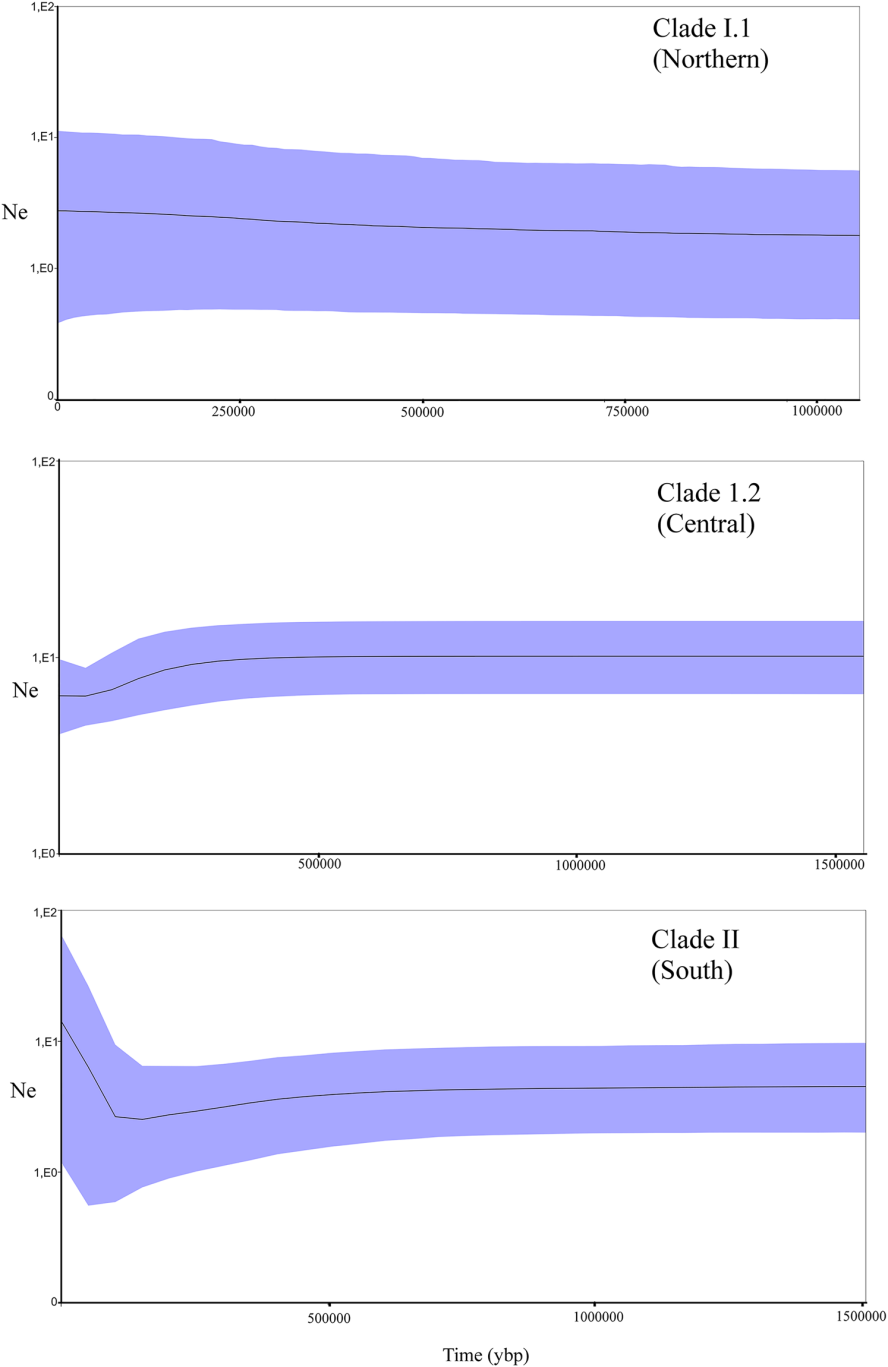
Bayesian Skyline Plots (BSP) representing demographic trends of the three main mitochondrial phylogroups of *Parastacus pugnax*. The blue area around the curve represents 95% confidence intervals for effective population size.

The results obtained from BSP were different for the three main clades and suggest a latitudinal pattern (Fig. 5). While population stability was suggested for the northern clade I.1, the other two clades show signs of population reductions, being that of clade II of greater magnitude. For the broadly distributed central clade (I.2) the reduction of effective population size (Ne) would have started about 300,000 ya, reaching a minimum about 60,000 ya and with no signal of recovery. Ne reductions for the southern clade (II) would have started about 500,000 ya showing signals of population size recovery after a minimum reached about 120,000 years ago. Fs statistic values suggest a higher frequency of population imbalance and recent expansions towards the south (Table 1). Of the 13 estimates of Fs that were significant and indicative of population reduction and posterior expansion, 10 were obtained from populations located from the Biobío basin and to the south.

### Current and historical distribution

**Figure 6:**
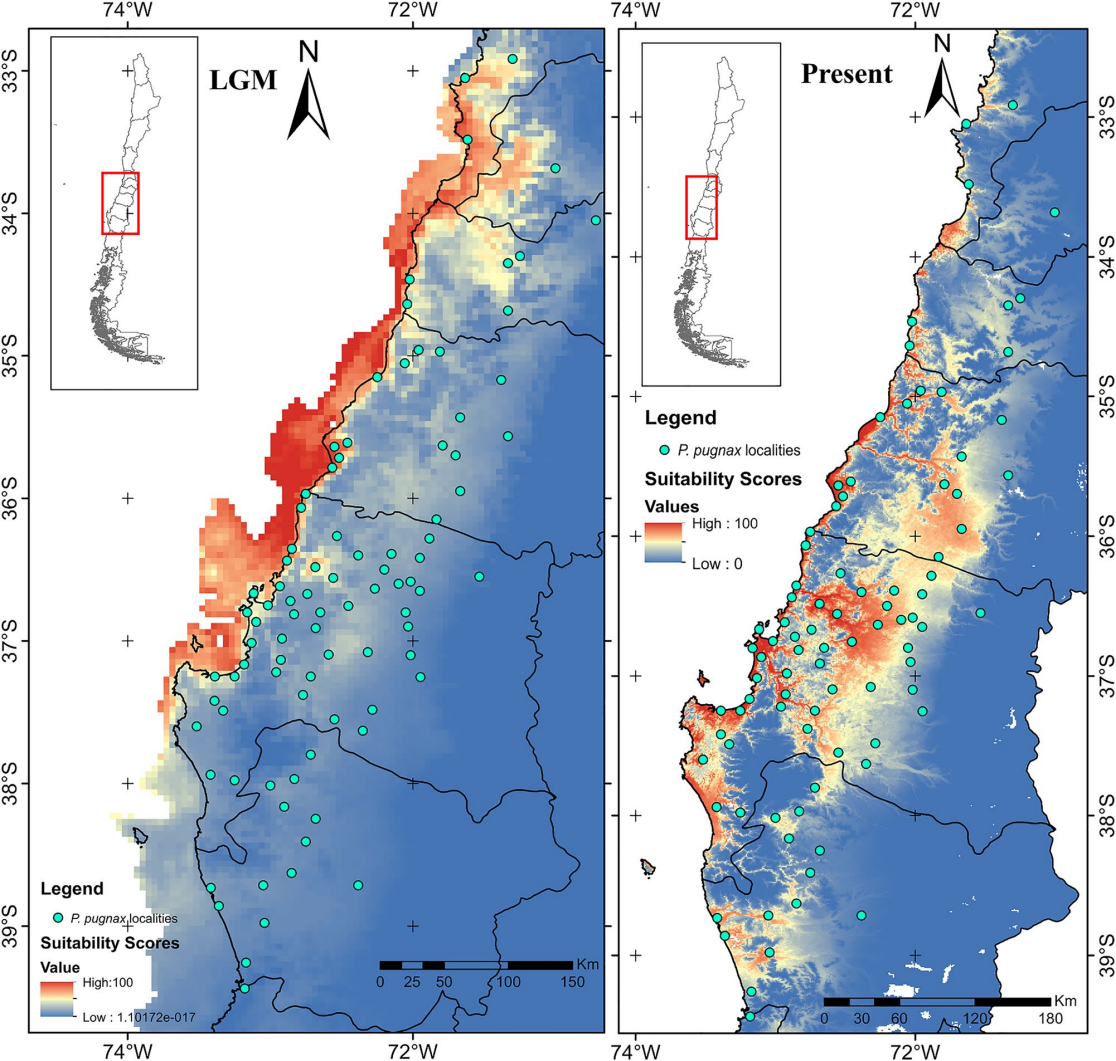
Habitat suitability map for *Parastacus pugnax* during the Last Glacial Maximum (LGM) and the present. The model and maps were generated with the software MaxEnt v.3.3.3e^75^ (http://biodiversityinformatics.amnh.org/open_source/maxent/).

Ecological niche modeling predicts a current distribution for *P. pugnax* from about 33° S, near the Pacific coast, to 39° S in Araucanía (Fig. 6). According to the bioclimatic and soil attributes of the habitat of this crayfish, the highest probabilities of occurrence were predicted for low areas and flat relief, where the largest distribution area is located in the extensive Intermediate Depression, across the Maule, Itata and part of the Biobío basins. This area has a relief with little undulation that extends between the Coastal range and the Andes, almost uninterruptedly, from 35° S to approximately 37°30′ S. High suitability scores were also obtained for areas following the course of the Andean rivers, which cross the coastal mountain range, connecting the Intermediate Depression with the narrow strip of lowland habitat that extends along the Pacific coast. At the coastal areas, the most extensive high suitable habitat encompasses the coastal plains of the Carampangue, Lebu and Paicaví basins, between 37° S and 38° S. Both in the northern coastal range (north of 34° S) and in the southern limit, suitable and isolated areas were predicted. Among these, an area of considerable extension is the one encompassing the Imperial basin and nearby coastal basins, close to 39° S. During the Last Glacial Maximum (LGM) *P. pugnax* would have shifted its distribution towards the west related to its present distribution, including extensive territories currently undersea that were emerged due to sea level dropped during the glaciations. During those times, the presence of *P. pugnax* in the Intermediate Depression would have considerably decreased, while stable habitats would have remained in the coastal basins. North of 34° S, there would have been an increase in the extension of habitat in non-coastal environments as well. Meanwhile, in the south, where clade II is distributed, a marked historical environmental instability was inferred.

## Discussion

The Chilean biota has been at the center of several phylogeographic studies. These have focused on marine e.g.,^33^ and continental species. Among the latter, there are studies on plants e.g.,^34^, invertebrates e.g.,^35^, and vertebrates e.g.,^36^, covering distinct life histories, such as aquatic and terrestrial forms and type of habitats (e.g., Valdivian forest specialists species)^15,17^. However, no study has focused on the specialists of a widely distributed type of habitats as are the subterranean waters (i.e., wet meadows or vegas) of central and south central Chile. This study is the second^30^ phylogeographic analysis of a Chilean species of *Parastacus*, a genus of South American endemic subterranean crayfishes. We found that *P. pugnax* is composed of lineages whose age precedes the uplift of the Andes; the old genetic lineages encompassed in this taxon would have arisen by the late Eocene. We also detected high levels of geographic structuring of the species genetic diversity, and we found signals of population size changes, which increased in magnitude towards the southern range of the species. Below we discuss these findings in detail.

Three lineages of ancient divergence were detected within *P. pugnax* (although several others, nested within the main three are also of considerable age), whose geographic boundaries could be explained by alluding to both climatic factors and relief (see Fig. S1). The most basal divergence of *P. pugnax*, which would have occurred about 38 million years ago, separated the lineage south of the Biobío basin (mainly the Imperial basin), from the rest of the populations from Biobío to the north. Phylogeographic breaks at a similar latitude have been reported for other taxa, such as the red cricket*, Cratomelus armatus*^35^, and the lizards *Liolaemus tenuis* and *L. pictus*^15,11^, with divergences that, in some cases, could warrant the recognition of different species. Given that the biology of such taxa is different, and the divergence times are much more recent than that of *Parastacus*, we suggest that the drivers of evolutionary differentiation at such latitude would have occurred recurrently, during the Cenozoic. This break is concordant with the bioclimatic transition between the Wet Mediterranean zone in the north and the wetter Valdivian zone in the south and one of the main biogeographic transition zones of Chile^37^, suggesting the confluence of several factors that have reduced the permeability of this complex of barriers, promoting the evolutionary differentiation at different taxonomic levels. At this latitude, the Intermediate Depression is interrupted by mountainous elevations, and three deep fluvial canyons that also interrupt the plane of the habitat of *P. pugnax*. The effects of the glaciations were of greater intensity towards the south of South America, where they also affected the low altitude areas. In this context, the effect of the glaciations, would have been larger at latitudes where the southernmost clade of *P. pugnax* is distributed^38^. Similarly, a synergistic effect of mountains and rivers, Cordillera Cantillana and Maipo River (Fig. S1), could have prompted the split of the northern clade of *P. pugnax*. These barriers have been already suggested as the drivers of the differentiation of lizards^20^.

According to previous phylogeographic studies, aquatic continental organisms tend to have their genetic diversity more structured than other organisms e.g.,^39^. This pattern of highly structured variation is consistent with those of other parastacids^40–42^, including *P. pugnax*. In general, we detect low genetic diversity and predominance of private haplotypes within each sampling locality, but high differentiation among them. We also detected a tendency to differentiate between basins (see AMOVA and Fst results), although the basins are not reciprocally monophyletic. Likely, events of river capture may explain the observed pattern of non-monophyletic basins^43^.

*P. pugnax* is a species with low vagility and direct development^28^, traits that likely contribute to mold the uncovered patterns of high genetic differentiation at a small geographical scale. Although there is a significant positive relationship in the analysis of isolation by distance (Fig. S2), in comparisons between nearby localities (less than 10 km) we found both low and high values of genetic differentiation. At the same time, our results show evidence of historical connections among different basins (e.g., the Andean basins of the Intermediate Depression). The greater historical connectivity among the Andean basins of the central clade (I.2) could be due to the flat relief that encompasses the boundaries among them (Fig. S1); in some places the boundary between the Itata and Maule basins is established by no more than 20 m of altitude. Considering the dynamic relief of a seismic as well as intensive orogenic country, it is likely that during the long history of *P. pugnax*, there were episodes of basin reconfiguration and connection/isolation that allowed gene flow among basins. Meanwhile, the historical connection of some Andean and coastal basins may have been prompted by sea level drops during glaciations (e.g., 130 m bellow current sea level during LGM)^44^; emerged land may have served to connect nearby but today disconnected basins that reach the ocean. The LGM-ENM model indicates that *P. pugnax* expanded into currently submerged environments, extending its range to the west of the current coastline. This scenario has been suggested to explain observed patterns of genetic variation of Australian^45^ and Argentinean^21^ fishes. However, the Chilean continental shelf is narrow, which would have made the connection between neighboring coastal basins unlikely, except for those associated with larger plains, such as Carampangue and Lebu, for which we found shared haplotypes. As such, our results are concordant with those found for the eastern Australian species of *Euastacus* that show high levels of genetic differentiation among a series of small coastal basins^40^.

The climatic changes associated with glaciations have produced considerable effects on the Chilean biota e.g.,^11,46–49^, where changes in distributional ranges and Ne would have been stronger at higher latitudes. Our results suggest that *P. pugnax* was affected by the glaciations, with the effect being larger south of the Biobío-Nahuelbuta (clade II). Although the ENM modeling suggests large habitat suitability changes for the LGM, the estimated reductions of Ne were not associated with the last glaciation, as they are much older. In South America, glaciations have been a cyclical process during the last 6 My^9^^.^ For *P. pugnax* the reductions in distribution would have been of greater magnitude in the interior valley of the Intermediate Depression than in coastal areas, which would not only have been climatically more stable, but also would have increased in extension as sea level decreases. However, we did not find clear signals of greater variability, expected for historically stable populations, in the coastal basins. This suggests additional factors, not considered here, that would have reduced coastal population sizes. One of these factors is likely basin size, as larger basins tend to harbor more genetic diversity (when considered as pi), which in a scenario of neutrality suggests that larger basins would harbor larger populations. In this regard is of interest to note the likely effect of repeated marine transgressions, to which the lowlands near the Pacific coast would have been more exposed, than the interior environments of the Andean basins.

The existence of ancient clades within *P. pugnax* suggests the existence of cryptic specific diversity that should be further analyzed with additional markers and the analysis of morphological variation. The existence of cryptic diversity in the western slope of the southern Andes, which is starting to be uncovered, seems to be widespread among taxonomic groups e.g.,^15,19,50,51^. Similarly, several studies have delimitated morphologically species in the family Parastacidae. For example, the existence of more than one species was recently proposed within the Australian *Cherax destructor*^52^. This suggests that parastacids, due to their age and biological attributes, would be prone to speciate without much morphological variation. The scarce exophenotypic differentiation in underground organisms, such as primary burrowing crayfishes, has been explained in terms of the restrictive and homogeneous conditions of the subterranean microhabitat, which would cause morphological stasis^53,54^. The little phenotypic variation displayed by some species of *Parastacus* is evident when comparing, for example, the species of Brazil and Chile, which would have diverged more than 40 Mya. A genealogical study suggested the existence of cryptic speciation in *P. brasiliensis*^42^^.^ In agreement with this, six new Brazilian species of *Parastacus* have been described in the last five years supported largely by genetic distinction^55–58^, which suggests that the real species richness of the genus is far from being fully detected. As such, the scenario that we are uncovering for the Chilean *P. pugnax* is not unexpected.

Noting that no taxonomic innovation is advanced in this study, if further studies corroborate that the three main mitochondrial lineages of *P. pugnax* uncovered here, correspond to three distinct species, some interesting taxonomic issues emerge. Two nominal forms are synonymized under *P. pugnax*. One is *P. chilensis* (Milne-Edwards, 1837) with type locality in Valparaiso, a locality lying in the distribution of the northern clade I.1. As such, the name *P. chilensis* is available for this lineage. The other taxon now synonymized under *P. pugnax*, is *P. hassleri* (Faxon, 1898) with type locality in Talcahuano, which is also the type locality of *P. pugnax*. As such *P. pugnax*, with *P. hassleri* in its synonymy, applies in a restricted sense to the large central clade (I.2). Remarkably, no name is available for the southern lineage (clade II); as such, if this lineage is to be considered at the species level, a new species should be described and named to encompass it.

Our results have relevant implications for the conservation of *P. pugnax *sensu lato. First, the genetic diversity of *P. pugnax* suggests the existence of several ESUs^26,59^ that should be treated as separate conservation units. For example, the southern clade II, although younger, presents three allopatric clades (i.e., Traiguén, Los Sauces, Imperial basin) that diverged more than 20 Mya. Several other clades are considerable old and also deserve conservation. In addition, the fact that almost each vega (locality) is monophyletic and possesses private haplotypes, confers high singularity on a small spatial scale. In this regard, special attention should be placed in the area of greatest extraction of the species, which corresponds to the range of the large central (I.2) and southern (II) clades. According to some statistics, more than 1500 ton are annually extracted, both during crayfish festivals and by people who use this species as a subsistence resource every winter^31^. From this point of view, *P. pugnax* is not only an evolutionary unique and genetically diverse species, but it is a valuable resource of cultural importance that is relevant to conserve.

This is the second phylogeographic work for *Parastacus* (see^30^) and provides evidence of great ancient diversity within one of the two currently recognized Chilean species of the genus. The uncovered phylogeographic pattern indicates an important role of the long-standing orogenic and climatic processes, which would have shaped the distribution of the high genetic diversity of the species, at the time that provides the basis for future studies integrating more genes and morphological evidence. Such studies would, for example, evaluate the distinction at the species level of the main lineages here uncovered. Our results are also a call of attention to ensure the conservation of the genetic diversity of *P. pugnax*, an intensely exploited resource.

## Methods

### Sample collection

We collected and preserved in 96% ethanol 465 crayfish specimens from 56 sampling localities in 17 river basins, each of which independently flow towards the Pacific Ocean. Of these, 12 basins were exclusively associated to the Coastal range (unconnected to Andean drainages) and five have headwaters in the Andes, running westwards and receiving distinct coastal drainages as tributaries before reaching the ocean (Fig. 1, Table 1). Sampling sites cover almost all the species range; our sampling misses the northernmost extreme of the distribution of *P. pugnax*. Specimens are housed in the Museum of Zoology of the University of Concepción, Chile. Crayfishes were sampled with the Servicio Agrícola y Ganadero (SAG-Chile) Permit Resolution N° 2687, and Bioethics Certificate VRID-Universidad de Concepción. Chile. Sampling sites were classified into three groups, corresponding to the main Chilean bioclimatic zones as follows (see^37^; acronyms in Fig. 1 and Table 1): (1) North, corresponding to the Sub-humid Mediterranean bioclimate zone, including localities from six river basins (NIL, MAT, PU, RE, BU, MAU); (2) Central, which corresponds to the Wet Mediterranean area, including five river basins (ITA, RAF, AND, THN, BIO); and (3) South, covering the Per-humid Mediterranean area, including six river basins (CO, CAR, LEB, PAI, IMP, Saa). In addition, we distinguish within each group the exclusively Pacific coastal basins and those of Andean origin.

### DNA sequence acquisition

DNA was extracted from muscle tissue (first pereiopod) using a commercial kit (Wizard SV Genomic; Promega) following the instructions of the manufacturer. The primers LCO-1490 (5′-GTCAACAAATCATAAAGATATTGG-3′) and HCO-2198 (5′-TAAACTTCAGGGTGACCAAAAAATCA-3)^60^ were used to amplify a fragment of the mitochondrial cytochrome c oxidase subunit I (COI) gene. Polymerase chain reactions (PCR) were performed in a total volume of 25 µL containing 12.5 µL Thermo Scientific PCR Master Mix, 0.2 µM of each primer and 20 ng of template DNA. PCR reactions were performed under the following conditions: an initial 2 min step at 95 °C, followed by 35 cycles with 45 s at 95 °C, 45 s at 50 °C, 1 min at 72 °C, and a final extension of 5 min at 72 °C. Amplicons were sequenced at Macrogen Inc., Seoul, Korea. Sequence editing was done using Codon Code Aligner v. 3.0.3 (Codon Code Corporation 2009); sequences were translated into amino acids in order to verify the absence of stop codons and reading frame shifts. New sequences were deposited into Genbank (MW135536-MW136000).

### Genetic diversity and genealogical analysis

Sequence alignment was done using Codon Code Aligner v. 3.0.3. The magnitude of nucleotide substitution saturation for the gene was tested using DAMBE software^61^. Descriptive statistics were calculated both for sampling localities and river basins. Genetic diversity was estimated in terms of the number of haplotypes (H), haplotype diversity (Hd), and nucleotide diversity (Pi) using Arlequin 3.5^62^. Average genetic divergence (including standard errors) among basins was estimated based on p-distances using Mega 6^63^_._ In order to estimate the association of geographic distance between sampling localities and genetic distance (p-distance), we estimated the correlation between both variables. This analysis was done in Primer v.7.0, with the Relate option^64^. To assess if there is an association between the basin size and genetic diversity (Hd and pi) a Spearman non-parametric correlation was performed using the option Relate in Primer v 7.0, with 10,000 permutations (https://www.primer-e.com/). This analysis correlates, using a Spearman's non-parametric method, hemimatrices of basin size and hemimatrices of genetic diversity values.

A median-joining (MJ) network^65^ of COI gene sequences was constructed to visualize the relationships among haplotypes using PopArt 1.7 (http://popart.otago.ac.nz). In addition, a gene tree was constructed using Bayesian inference (BI) in MrBayes 3.0b4^66^. We added sequences of the Brazilian *P. brasiliensis* (KU258636) and *P. pilimanus* (FJ966039) to root the tree, assigning it the condition of sister species of *P. pugnax*^25^. The Akaike’s information criterion (AIC) and Bayesian information criterion (BIC)^67^ implemented in jModelTest 2^68^ indicated the T92 + G + I as the best-fit model for the COI data. Two independent Bayes runs during 6,000,000 generations each were performed and sampled every 1000 generations. Each run consisted of four Monte Carlo Markov chains (MCMC). After discarding the first 15,000 samples as burn-in, the remaining trees were used to create a 50% majority rule consensus tree with posterior probability (PP) values.

### Estimating divergence times

In order to estimate divergence times of the main clades of *P. pugnax*, the nucleotide substitution rate for the Cytochrome Oxidase I (COI) region of the Parastacidae family was obtained with BEAST v1.8.3^69^ using COI gene sequences of 91 species from South America, Madagascar, New Zealand, and Australia, obtained from Genbank, plus samples of the Parastacidae family from Chile obtained by us in this study (Table S1). We also included representatives of the superfamilies Astacoidea, Nephropoidea, and Callianassoidea, which were used to form the outgroup.

A strict molecular clock at species level was calibrated using the first fossil appearance of the families Parastacidae, Chimerastacidae, Astacidae, and Cambaridae (see details in Table 2 of [27]). The analysis was carried out with a YuleBirth-Death process speciation model with a HKY + G + I model (selected through jModeltest^70^,*P. pugnax*,*Parastacus varicosus*,*P. pilimanus*,*P. brasiliensis*,*P. defossus*,*Virilastacus araucanus*,*V. retamali*,*V. rucapihuuelensis*,*V. jarai*,*Samastacus spinifrons*,*Paranephrops planifrons*,*Cherax parvus*; the latter two were used to form the outgroup (Table S1,^71^ in order to see the distribution of the parameters and the quality of the likelihood probability to then obtain the consensus tree with TreeAnnotator v1.8.3^71^,http://tree.bio.ed.ac.uk/software/figtree/).

#### Genetic Structure

A spatial analysis of molecular variance (SAMOVA 2.0; cmpg.unibe.ch/software/samova2) was conducted to find spatially homogeneous and highly differentiated locality groups^72^. We tested values for K in the range of 2–17 (according to the maximum number of basins); starting condition was set to 100 with 10,000 iterations. The best configuration of population groups, which was the one retained, show the highest Fct value and less than 50% of clusters composed of a single population^72^.

In addition, we inferred the presence of historical genetic barriers among the populations of *P. pugnax* using Monmonier’s maximum difference algorithm implemented in BARRIER 2.2^73^. BARRIER uses a triangulation network to connect contiguous populations by means of a Voronoi tessellation set to detect probable barriers to gene flow among differentiated populations^73^. Each edge of the Voronoi polygons was associated with the value of the corresponding Fst value between pairs of populations, calculated with ARLEQUIN. The method highlights the limits of the Voronoi tessellation associated with the highest values of Fst between pairs of populations, thus identifying multiple barriers in the hierarchical progression of Fst values. We only considered genetic discontinuities greater than the general average of the Fst between all population pairs.

A hierarchical analysis of the spatial arrangements of genetic diversity of *P. pugnax* was conducted by an analysis of molecular variance (AMOVA)^74^ using Arlequin. Haplotypes were grouped by sampling localities and basins.

### Historical demography

In order to assess patterns of historical demographic changes, we estimated the departure from neutrality statistic Fs (Fu, 1997), as implemented in Arlequin 3.5. This index was estimated for each locality, as well as for each basin. Similarly, historical population sizes were estimated for each main clade of *P. pugnax* using the Bayesian Skyline Plot (BSP) method implemented in BEAST^75^. Piecewise-constant Skyline Model was selected, and a relaxed uncorrelated lognormal molecular clock was applied with the mutation rate obtained from our calibration (3% per My). We ran 10,000,000 generations and discarded the first 10% as burnin. Distributions were used to generate confidence intervals corresponding to the uncertainty of the coalescence process^76^; we used Tracer 1.7^77^ to monitor parameters estimates and ESS values.

### Present and past distribution modelling

To estimate the potential current distribution and identify probable refugia during the LGM, environmental niche models (ENMs) were generated with Maxent 3.3.3e^78^. We downloaded contemporary (1950–2000) and LGM (~ 21Ka) climate data at a 2.5 arc-minutes resolution (nearly 5 × 5 km) from WorldClim^79^. Additionally, a more accurate (1 km resolution) distribution model was generated for the present using elevation data from the NASA Shuttle Radar Topography Mission (SRTMv4.1)^80^ and bioclimatic variables from WorldClim. Furthermore, given the ecological features of *P. pugnax*, we used soil parameters at 1 km resolution and 1 m depth, as bulk density, clay content, volumetric percentage of coarse fragments, silt content, sand content, soil pH, and soil organic carbon stock^81^. Pearson’s correlation coefficients were estimated for each pairwise comparison of variables using ENM Tools v 1.4.3^82^; highly correlated variables (Pearson’s coefficient 0.9) were removed from analysis. We performed a priori model testing to determine optimal combinations of the regularization and feature parameters for the construction of ENMs^83^. We tested model combinations of regularization parameters from 1 to 10 in intervals of 1 and feature classes. Model performance under different combinations of these settings was evaluated using AIC as optimality criterion to protect from overfitting. The R package ENMeval v 0.1.1 (http://cran.r-project.org/web/packages/ENMeval^84^; was used to obtain models and jackknife replicates. We estimated average values across k iterations for ORMTP, OR10, and AUCTEST (AUC based on estimated values for localities). The combination of feature class (FC) and regularization multiplier (RM) was selected for subsequent analyses.

## Supplementary Information

Supplementary Information.

## Data Availability

New DNA sequences are deposited in Genbank with numbers MW135536-MW136000.
