# Supplementary material for: Evolving in islands of mud: old and structured hidden diversity in an endemic freshwater crayfish from the Chilean hotspot
Source: Sci Rep. 2021 Apr 21;11:8573. doi: 10.1038/s41598-021-88019-8 (PMC8060301; doi:10.1038/s41598-021-88019-8)
Supplement: Supplementary file 1 — Supplementary Information. [file 41598_2021_88019_MOESM1_ESM.docx]

**SUPPLEMENTARY MATERIAL**

**Evolving in islands of mud: old and structured hidden diversity in an endemic freshwater crayfish from the Chilean hotspot**

Pedro Victoriano and Guillermo D’Elía

Figure S1. Topographic units and limits of bioclimatic zones throughout the distribution of *Parastacus pugnax*. Adapted from Muñoz-Mendoza et al. (2017). The map was generated with QGIS v.3.16.2 (https://qgis.org/es/site/) and modified using Inkscape v.0.91 (http://inkscape.org/).

**
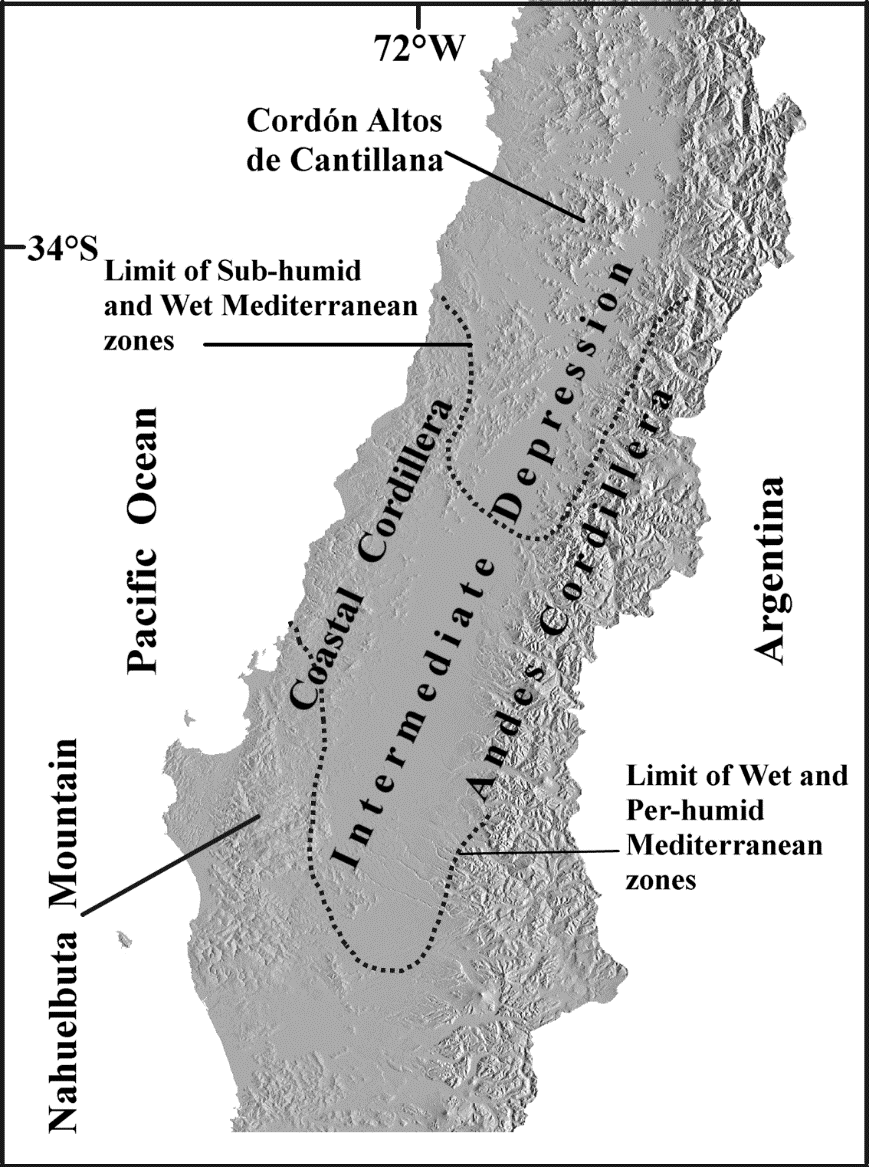
**

Figure S2. Association for p-distance v/s geographical distance for locality-pairs of *Parastacus pugnax*. Rho (Spearman) = 0.239; p = 0.008 (0.08%).


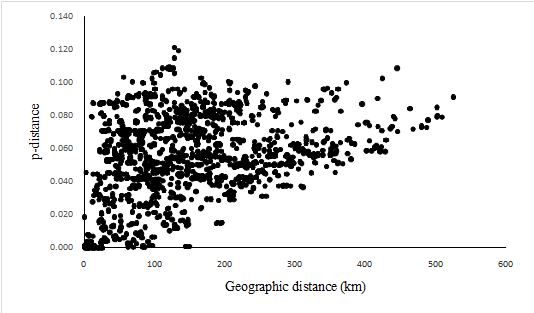


Figure S3. Correlation between basin size and nucleotide diversity (pi) of local samples of of *Parastacus pugnax*. Rho (Spearman) = 0.621; p <0.001;


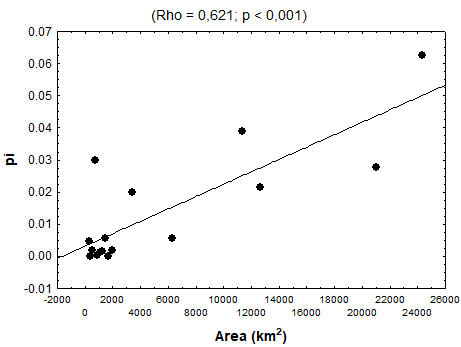


Table S1. Sequences obtained from GenBank used to calculate the substitution rate of the COI gene for the family Parastacidae; see text for details.

| **Genus** | **Species** | **GenBank accession #** |
| --- | --- | --- |
| *Astacopsis* | *gouldi* | DQ006289 |
| *Astacopsis* | *tricornis* | DQ006290 |
| *Cherax* | *albidus* | FJ965956 |
| *Cherax* | *boesemani* | KU821416 |
| *Cherax* | *cairnsensis* | EU921142 |
| *Cherax* | *crassimanus* | AF493625 |
| *Cherax* | *cuspidatus* | DQ006292 |
| *Cherax* | *dispar* | FJ965957 |
| *Cherax* | *gherardii* | KU821418 |
| *Cherax* | *glaber* | FJ965958 |
| *Cherax* | *holthuisi* | KU821421 |
| *Cherax* | *leckii* | KT454706 |
| *Cherax* | *parvus* | DQ006293 |
| *Cherax* | *peknyi* | KU821423 |
| *Cherax* | *preissi* | AF493623 |
| *Cherax* | *pulcher* | KU821424 |
| *Cherax* | *quadricarinatus* | DQ006294 |
| *Cherax* | *quinquecarinatus* | FJ965959 |
| *Cherax* | sp. | KP697509 |
| *Cherax* | *tenuimanus* | AF493631 |
| *Tenuibranchiurus* | *glypticus* | FJ965973 |
| *Engaeus* | *cunicularius* | HG942173 |
| *Engaeus* | *fossor* | EU921144 |
| *Engaeus* | *lyelli* | HG799086 |
| *Engaeus* | *structifrons* | AF493633 |
| *Engaeus* | *sericatus* | FJ965960 |
| *Engaewa* | *reducta* | FJ965971 |
| *Euastacus* | *armatus* | FJ965962 |
| *Euastacus* | *australasiensis* | FJ965963 |
| *Euastacus* | *balanensis* | DQ006302 |
| *Euastacus* | *bidawalus* | DQ006315 |
| *Euastacus* | *bindal* | DQ00631 |
| *Euastacus* | *bispinosus* | AF493634 |
| *Euastacus* | *brachythorax* | DQ006319 |
| *Euastacus* | *clarkae* | DQ00632 |
| *Euastacus* | *crassus* | DQ006326 |
| *Euastacus* | *dangadi* | DQ006328 |
| *Euastacus* | *diversus* | DQ006331 |
| *Euastacus* | *dharawalus* | DQ006330 |
| *Euastacus* | *eungella* | EU921002 |
| *Euastacus* | *fleckeri* | DQ006337 |
| *Euastacus* | *gamilaroi* | DQ006339 |
| *Euastacus* | *gumar* | DQ006341 |
| *Euastacus* | *guwinus* | DQ006345 |
| *Euastacus* | *hystricosus* | DQ006348 |
| *Euastacus* | *jagara* | DQ006350 |
| *Euastacus* | *kershawi* | DQ006351 |
| *Euastacus* | *maidae* | DQ006354 |
| *Euastacus* | *monteithorum* | DQ006357 |
| *Euastacus* | *mirangudjin* | DQ006356 |
| *Euastacus* | *neohirsutus* | DQ006362 |
| *Euastacus* | *pilosus* | KT454688 |
| *Euastacus* | *polysetus* | DQ006363 |
| *Euastacus* | *reductus* | DQ006365 |
| *Euastacus* | *rieki* | DQ006367 |
| *Euastacus* | *robertsi* | DQ006378 |
| *Euastacus* | *setosus* | DQ006380 |
| *Euastacus* | *spinichelatus* | DQ006383 |
| *Euastacus* | *spinifer* | DQ006390 |
| *Euastacus* | *sulcatus* | DQ006396 |
| *Euastacus* | *suttoni* | DQ006398 |
| *Euastacus* | *valentulus* | DQ006402 |
| *Euastacus* | *woiwuru* | DQ006403 |
| *Euastacus* | *yanga* | DQ006409 |
| *Euastacus* | *yarraensis* | DQ006412 |
| *Euastacus* | *yigara* | DQ006414 |
| *Geocharax* | *gracilis* | EU921145 |
| *Geocharax* | *falcata* | AF493632 |
| *Gramastacus* | *insolitus* | FJ965961 |
| *Spinastacoides* | *insignis* | FJ965966 |
| *Ombrastacoides* | *huonensis* | EU921143 |
| *Paranephrops* | *planifrons* | DQ006415 |
| *Paranephrops* | *zealandicus* | DQ006416 |
| *Astacoides* | *madagascariensis* | FJ965964 |
| *Astacoides* | *crosnieri* | EU921147 |
| *Astacoides* | *betsileoensis* | EU921146 |
| *Astacoides* | *caldwelli* | FJ965965 |
| *Parastacus* | *brasiliensis* | EF599158 |
| *Parastacus* | *defossus* | FJ965968 |
| *Parastacus* | *pilimanus* | FJ965967 |
| *Parastacus* | *pugnax* | EF599157 |
| *Parastacus* | *varicosus* | FJ965969 |
| *Samastacus* | *spinifrons* | EF599159 |
| *Virilaastacus* | *jarai* | FJ965970 |
| *Virilastacus* | *araucanius* | EF599156 |
| *Virilastacus* | *retamali* | EF599155 |
| *Virilastacus* | *rucapihuelensis* | EF599149 |
| *Astacus* | *astacus* | AF517104 |
| *Pacifastacus* | *leniusculus* | EU921148 |
| *Cambarellus* | *schufeldtii* | EU921149 |
| *Orconectes* | *virilis* | AF474365 |
| *Procambarus* | *clarkii* | AY701195 |
| *Homarus* | *americanus* | DQ889104 |
| *Sergio* | *mericeae* | FJ965972 |

Table S2. Genetic distance (*p*-values: bellow the diagonal; Standard Deviation: above diagonal) for pairwise comparisons between basins, throughout the distribution of *Parastacus pugnax*. Basin codes are as in Table 1. Basin are ordered latitudinally (North to the left). Green: lowest values; red: highest values.
